# Accelerated activation of the coagulation pathway during cardiopulmonary bypass in aortic replacement surgery: a prospective observational study

**DOI:** 10.1186/s13019-015-0295-9

**Published:** 2015-06-23

**Authors:** Hideo Sato, Koji Yamamoto, Akihito Kakinuma, Yoshinori Nakata, Shigehito Sawamura

**Affiliations:** 1 Department of Anesthesia, Teikyo University School of Medicine, 2-11-1 Kaga, Itabashi-ku, Tokyo, 173-8605 Japan; 2 Department of Transfusion Medicine and Cell Therapy, Saitama Medical Center, Saitama Medical University, Kawagoe, Saitama Japan; 3 Teikyo University Graduate School of Public Health, Tokyo, Japan

**Keywords:** Tissue factor, Activated factor VII, Cardiopulmonary bypass, Aortic replacement surgery, Extrinsic coagulation pathway

## Abstract

**Background:**

Any form of surgery or tissue damage causes release of tissue factor into the circulation. This may lead to the accelerated consumption of coagulation factors, resulting in severe consumptive coagulopathy. In this study, we compared the molecular markers involved in coagulation activation during cardiopulmonary bypass (CPB) between patients who underwent aortic replacement surgery and those who underwent valve surgery.

**Methods:**

This prospective observational study was performed in each 14 patients who underwent aortic replacement surgery or valve surgery. We evaluated the differences in the levels of fibrinogen, activated factor VII (FVIIa), thrombin–antithrombin complex (TAT), and soluble fibrin monomer complex (SFMC) during surgery between these two groups.

**Results:**

The change in fibrinogen levels showed no difference between the groups. The magnitude of increase in TAT was much larger in patients who underwent aortic replacement surgery than in those who underwent valve surgery (173.6 *vs.* 49.4 ng/mL; *p* = 0.0001). More importantly, the elevation of FVIIa was significantly higher in patients who underwent aortic replacement (28.5 *vs.* 19.0 mU/mL; *p* = 0.0122). The magnitude of increase in SFMC was also larger in the aortic replacement surgery.

**Conclusions:**

The activation of coagulation during CPB was dramatically higher in the aortic replacement surgery compared with the valve surgery, probably owing to the activation of the extrinsic coagulation pathway in the former. This could potentially exacerbate consumptive coagulopathy after CPB termination in patients who underwent aortic replacement, possibly resulting in massive hemorrhage due to impaired hemostasis.

## Background

Cardiovascular surgery is frequently accompanied by a bleeding tendency, probably resulting from the impairment of platelet activation and coagulation caused by cardiopulmonary bypass (CPB). Aortic replacement surgery using CPB is frequently complicated by massive hemorrhage that in turn, is most commonly aggravated by severe hypofibrinogenemia due to dilutional coagulopathy. It has been reported that not only dilutional coagulopathy but also consumptive coagulopathy by continuous thrombin generation progresses during CPB despite full heparinization of blood [1, 2]. The activation of the intrinsic coagulation pathway including the contact phase has been regarded to be more important in thrombin generation during CPB because of the direct contact of circulating blood with CPB. However, the activation of the extrinsic coagulation pathway by tissue factor (TF) released into the circulation may be a primary origin of the thrombin generation during CPB [3]. TF, which is abundantly expressed in the vascular wall, epicardium, fat, bone, lungs, brain, and muscles [4, 5], activates factor VII (FVII) and forms the TF/activated factor VII (FVIIa) complex. This complex may then generate thrombin massively during CPB despite full heparinization because of the limited thrombin-inhibiting effect and the subsequent soluble fibrin formation of heparin with antithrombin on the extrinsic coagulation pathway.

In general, aortic replacement surgery is so invasive that it can potentially cause widespread tissue damage and increase the release of TF into the circulation. So far, some studies have shown the activation of extrinsic coagulation pathway during CPB in coronary artery bypass grafting (CABG) or valve replacement surgery. However, the pathology and mechanisms of consumptive coagulopathy during CPB in aortic replacement surgery have not been elucidated [6]. In this study, we analyzed and compared some molecular markers of coagulation activation {e.g., thrombin-antithrombin complex (TAT), soluble fibrin monomer complex (SFMC), FVIIa} during CPB between patients undergoing aortic replacement surgery and those with valve surgery. Significantly higher levels of TAT and FVIIa during CPB were observed in patients who underwent aortic replacement surgery compared with those who underwent valve surgery. This observation suggests the accelerated activation of coagulation and consumptive coagulopathy, through the activation of the extrinsic coagulation pathway in patients who underwent aortic replacement, possibly resulting in the impaired hemostasis frequently observed in aortic replacement surgery.

## Methods

The Ethics Committee of the Teikyo University School of Medicine approved this study. We enrolled patients older than 20 years who were scheduled to undergo elective aortic replacement surgery or valve replacement surgery, including those undergoing surgery combined with CABG. With a significant level of 5 % and a power of 80 %, a total of 20 patients were needed to detect a 200 ng/ml difference in TAT level with a standard deviation of 150 ng/ml. We estimated that 30 patients should be included in the trial, 15 patients in each group, because of exclusion criteria and complications during surgery. Written Informed consent was obtained from all patients. From June 2013 until April 2014, 14 patients who underwent aortic replacement surgery and 14 patients who underwent valve replacement surgery were registered for this prospective observational study. Patients with congenital bleeding tendency or low platelet count (i.e., <100 × 10^3^/μL) were excluded. Patients who were administered anticoagulant or antiplatelet agents were enrolled if these medications were stopped before surgery. Patients with significant liver and/or renal dysfunction were excluded as well.

Anesthesia was induced with fentanyl (4–6 μg/kg), midazolam (2–5 mg/body), and rocuronium (0.6–1.0 mg/kg), and then, sevofulrane was used for maintenance. The patients received artificial respiration, with oxygen concentration at 40 % and end tidal carbon dioxide maintained at 30–40 mmHg. A peripheral intravenous line, a radial or a brachial arterial line, a central venous catheter, and the Swan-Ganz catheter were used to monitor the patients. Heparin at 300 U/kg was injected before the start of CPB, and additional heparin was suitably administered so that the activated clotting time would be more than 400 s throughout the procedure. Heparin was neutralized with protamine (3 mg/kg) at the end of CPB. A heparin coating or macromolecule polymer coating was used in all CPB circuits. Blood from the pleural and pericardial cavities were recirculated through the reservoir by suction. The transfusion in each case was decided in charge by the anesthesiologist because we do not have a standard protocol.

Blood samples were taken through arterial catheterization at the induction of anesthesia, at the start of CPB, 1 h from the start of CPB, and at the end of CPB (i.e., after protamine administration). We measured fibrinogen, SFMC, and FVIIa by using STA-R Evolution (Diagnostica STAGO, Asnières sur Seine, France), while TAT was evaluated by using STACIA (LSI Medience, Tokyo, Japan). As a reagent, STA® Fibrinogen (Diagnostica STAGO) was used for fibrinogen, STACIA CLEAIR TAT (LSI Medience) for TAT, Auto LIA®FM (Roche Diagnostics K.K., Tokyo, Japan) for SFMC, and STACLOT® VIIa-rTF (Diagnostica STAGO) for FVIIa.

We reported the data as the mean ± standard deviation or as the median (first quartile – third quartile) value. We used the unpaired *t*-test or Mann-Whitney *U* test to identify differences in the demographic data of patients. The Mann-Whitney *U* test was used to determine significant differences in the levels of the indicated markers during CPB between the groups. A *p*-value of <0.05 was considered statistically significant. Microsoft Excel Statistics 2012 was used for the statistical analysis.

## Results

The patient characteristics and preoperative laboratory data showed no significant differences between the groups, except that the TAT and SFMC levels were significantly higher in the aortic replacement surgery group (Table 1). No significant differences were also observed between the groups except the duration of CPB time, time required for hemostasis, bleeding and transfusion volumes during surgery (Table 2).

**Table 1:** Demographic data of patients

	Aortic Replacement Group (*n* =14)		Valve Replacement Group (*n* = 14)		*p* value
Age (years)	68.4 ± 6.5		70.8 ± 7.3		0.1809
Sex (M/F)	9/5		10/4		
Height (cm)	164.3 ± 10.6		158.9 ± 10.3		0.0946
Weight (kg)	64.8 ± 16.8		57.6 ± 11.3		0.0961
BSA (m^2^)	1.70 ± 0.24		1.59 ± 0.19		0.0892
Cases	Ascending Aorta Replacement + AVR	2	MVP	1	
			AVR	2	
	Descending Aorta Replacement + CABG	1	AVR + TAP	1	
	Descending Aorta Replacement	2	AVR + MVP	2	
	TAR + AVR	1	AVR + MVP + CABG	1	
	TAR + CABG	3	AVR + MVR + TAP	1	
	TAR	4	AVR + CABG	5	
	Ascending Aorta Replacement + AVR + CABG	1	MVR + TAP	1	
Fibrinogen (mg/dL)	339 (274–386)		314 (287–356)		0.4762
TAT (ng/mL)	7.7 (4–14.4)		2.1 (1.2–3.2)		0.0009*
SFMC (μg/mL)	20.8 (4.1–109.2)		3.9 (2.4–4.8)		0.0038*
FVIIa (mU/mL)	35.0 (26.3–42.3)		29.5 (21.3–34.8)		0.4616
Hemoglobin (g/dL)	11.6 (10.5–12.9)		11.1 (10.1–13.2)		0.5812
Platelet (×10^3^/mL)	168 (138–194)		169 (133–190)		0.7304
PT-INR	1.17 (1.11–1.24)		1.17 (1.14–1.21)		0.9084
APTT (s)	33.6 (29.8–35.1)		34.0 (29.9–37.0)		0.6458

AVR aortic valve replacement, MVP mitral valve plasty, MVR mitral valve replacement

TAP tricuspid annuloplasty, CABG coronary artery bypass grafting, TAR total arch replacement

*Significant difference between the two groups

**Table 2:** Intraoperative and postoperative data

	Aortic replacement group (*n* = 14)	Valve replacement group (*n* = 14)	*p* value
Heparin dose for CPB (×10^3^ IU)	18.0 ± 5.2	17.6 ± 3.8	0.4182
CPB time (min)	216 (181–232)	142 (112–179)	0.0024*
Duration of aortic crossclamp (min)	136 (110–182)	120 (86–153)	0.4479
Time required for hemostasis	77 (61–147)	42 (36–68)	0.0082*
Bleeding volume during surgery (mL)	3080 (1888–4539)	1535 (1204–1799)	0.0009*
Postoperative blood loss-24 h (mL)	1228 (611–1648)	746 (459–1519)	0.4622
Transfusion of red cell concentrates during surgery (U)	14 (10.5–17.5)	9 (4.5–14)	0.0421*
Transfusion of fresh frozen plasma during CPB (U)	6 (4–6)	0 (0–4)	0.0012*
Transfusion of fresh frozen plasma during surgery (U)	10 (7–20)	2 (0–7.5)	0.0023*
Transfusion of platelet concentrates during surgery (U)	20 (20–20)	0 (0–20)	0.0014*
Postoperative hemoglobin (g/dL)	10.9 (10.1–11.6)	11.1 (9.8–12.2)	0.8721

*Significant difference between the two groups

**Fig. 1:**
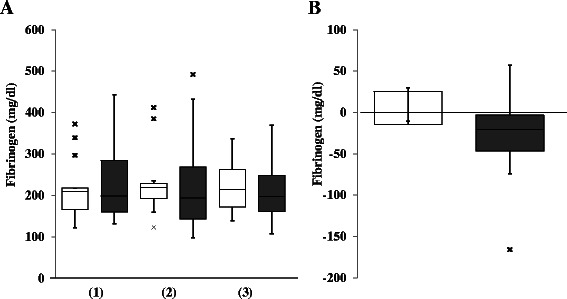
Comparison of fibrinogen level between the groups. **a**: Fibrinogen level at the start of cardiopulmonary bypass (CPB) (1), at 1 h from the start of CPB (2), and at the end of CPB (3). **b**: The change of fibrinogen level during CPB. □ (open column): valve surgery; ■ (closed column): aortic replacement surgery; x: outlier. Data are showed as box and whisker plots (25 and 75 percentiles, outliers). Outliers are showed as cases with values more than 1.5 times of box lengths from either end of the box.　No significant difference in the change of fibrinogen level during CPB was detected between the groups

As it has been suggested that the bleeding tendency and impaired hemostasis during surgery depends upon the decrease in the plasma level of fibrinogen, we measured the fibrinogen level during CPB in each case to evaluate the progression of coagulopathy. Although we observed the tendency for hypofibrinogenemia progression during CPB in the aortic replacement surgery group, the magnitude of decrease in the fibrinogen level during CPB was not significantly different between the groups (Fig. 1). We transfused fresh frozen plasma (FFP) before termination of CPB as a conventional therapy to prevent severe hypofibrinogenemia. A median of 6 [3–5] units was transfused in 12 patients who underwent aortic replacement, and a median of 0 (0–4) units was transfused in 5 patients who underwent valve surgery, showing a significant difference between the groups (*p* = 0.0012).

**Fig. 2:**
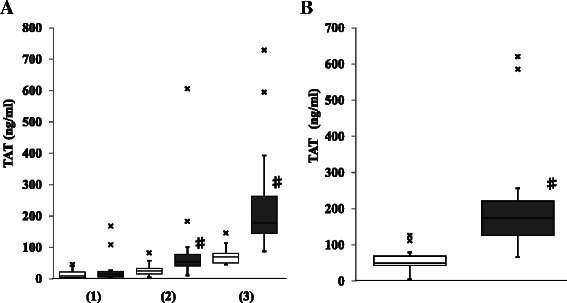
Comparison of thrombin–antithrombin complex (TAT) between the groups. **a**: TAT at the start of cardiopulmonary bypass (CPB) (1), at 1 h from the start of CPB (2), and at the end of CPB (3). **b**: The increase in TAT during CPB. □ (open column): valve surgery; ■ (closed column): aortic replacement surgery; x: outlier; Data are showed as box and whisker plots (25 and 75 percentiles, outliers). Outliers are showed as cases with values more than 1.5 times of box lengths from either end of the box.　#: *p* < 0.05

**Fig. 3:**
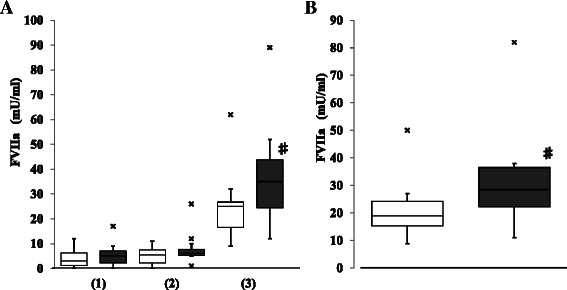
Comparison of activated factor VII (FVIIa) between the groups. **a**: FVIIa at the start of cardiopulmonary bypass (CPB) (1), at 1 h from the start of CPB (2), and at the end of CPB (3). **b**: The increase in FVIIa during CPB. □ (open column): valve surgery; ■ (closed column): aortic replacement surgery; x: outlier; Data are showed as box and whisker plots (25 and 75 percentiles, outliers). Outliers are showed as cases with values more than 1.5 times of box lengths from either end of the box. #: *p* < 0.05

To examine the activation of coagulation during surgery, the levels of TAT and FVIIa during CPB were measured in each case. Although the TAT level at the start of CPB showed no difference between the groups, it dramatically increased during CPB in the aortic replacement group (Fig. 2a). The TAT levels at 1 h from the start of CPB and at the end of CPB were significantly higher in the aortic replacement group than those in the valve surgery group (i.e., 54.4 [40.8–76.9] ng/mL *vs.* 24.3 [14.9–33.6] ng/mL, *p* = 0.0051 at 1 h of CPB; 178.8 [144.2–262.5] ng/mL *vs.* 70.3 [50.6–80.4] ng/mL, *p* = 0.0001 at the end of CPB). Therefore, a significantly larger increase in the TAT level during CPB was demonstrated in the aortic replacement group than that in the valve surgery group (i.e., 173.6 [125.6–221.5] ng/mL *vs.* 49.4 [42.7–69.2] ng/mL, *p* = 0.0001) (Fig. 2b). Meanwhile, both the FVIIa level at the end of CPB and the increase in the FVIIa during CPB were significantly higher in the aortic replacement group than in the valve surgery group (i.e., 35 [24.5–43.8] mU/mL *vs.* 25 [16.5–26.8] mU/mL, *p* = 0.0289 at the end of CPB; 28.5 [22.3–36.5] mU/mL *vs.* 19.0 [15.3–24.3] mU/mL; *p* = 0.0122 during CPB) (Fig. 3). Thus, accelerated coagulation activation progressed via the extrinsic pathway during CPB in the aortic replacement surgery group compared with the valve surgery group.

**Fig. 4:**
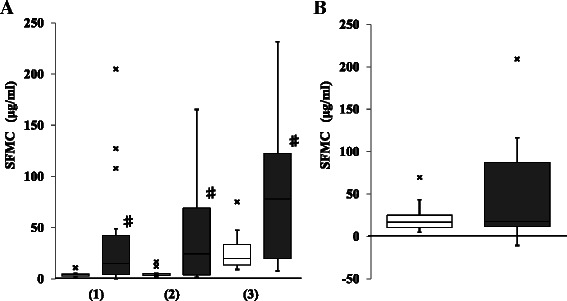
Comparison of soluble fibrin monomer complex (SFMC) between the groups. **a**: SFMC at the start of cardiopulmonary bypass (CPB) (1), at 1 h from the start of CPB (2), and at the end of CPB (3). **b**: The increase in SFMC during CPB. □ (open column): valve surgery; ■ (closed column): aortic replacement surgery; x: outlier; Data are showed as box and whisker plots (25 and 75 percentiles, outliers). Outliers are showed as cases with values more than 1.5 times of box lengths from either end of the box. #: *p* < 0.05. No significant difference in the increase in SFMC during CPB was detected between the groups

**Fig. 5:**
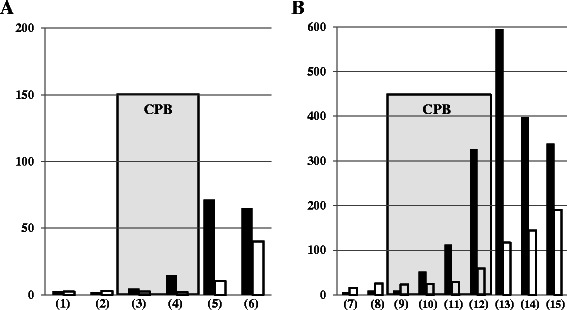
Time courses of coagulation markers during surgery in the representative case of each group. **a**: valve surgery; **b**: aortic replacement surgery. ■ (closed column): thrombin–antithrombin complex (ng/mL); □ (open column): soluble fibrin monomer complex (μg/mL). CPB time is shown by the gray zone. (1) and (7): pre-operation; (2) and (8): pre-CPB; (3) and (9): the start of CPB; (4) and (10): 1 h after the start of CPB; (5) and (13): the end of CPB; (6) and (15): post-operation; (11): 2 h after the start of CPB; (12): 3 h after the start of CPB; (14): 1 h after the end of CPB

Finally, we analyzed the level of SFMC, which shows the degree of fibrin generation and consumption of fibrinogen, during CPB in each case. We observed significantly higher levels of SFMC at all measurement time points in the aortic replacement group than those in the valve surgery group (i.e., 15.0 [4.2–42.5] μg/mL *vs.* 4.1 [3.0–4.8] μg/mL, *p* = 0.0179 at the start of CPB; 24.5 [3.9–69.1] μg/mL *vs.* 3.9 [3.5–5.2] μg/mL, *p* = 0.0344 at 1 h of CPB; 78 [20–122.6] μg/mL *vs.* 19.8 [13.5–33.6] μg/mL, *p* = 0.0216 at the end of CPB) (Fig. 4a). Although the magnitude of increase in SFMC levels during CPB was larger in the aortic replacement surgery group, no significant difference was observed between the groups (Fig. 4b). To demonstrate the typical accelerated activation of coagulation in aortic replacement surgery compared with valve surgery, the time courses of fibrinogen, TAT, and SFMC in the representative case for each group are shown in Fig. 5. The aortic replacement surgery case showed a dramatic elevation of TAT and SFMC during CPB. A total of 720 mL of fresh frozen plasma was transfused to prevent progressing hypofibrinogenemia, resulting in diminished reduction of the fibrinogen level (Fig. 5b).

## Discussion

Cardiovascular surgery using CPB decreases the plasma concentration of coagulation factors, including fibrinogen, primarily by hemodilution with CPB priming and intravenous fluids [7]. Thoracic aortic replacement surgery is frequently accompanied by massive hemorrhage due to severe hypofibrinogenemia [8]. A recent report showed that fibrinogen concentration frequently fell below 150 mg/dL during CPB in patients who underwent thoracic aortic repair surgery [8]. We also observed the tendency for hypofibrinogenemia progression during CPB in the aortic replacement group, but not in the valve surgery group (Fig. 1). In order to avoid massive bleeding due to severe hypofibrinogenemia, we usually transfuse FFP before termination of CPB in aortic replacement surgery. Indeed, significantly more number of units of FFP was transfused during CPB in the aortic replacement group, implying that the fibrinogen levels tended to decrease more during CPB in aortic replacement surgery compared with valve surgery. Although there were no significant differences in the decrease in plasma fibrinogen level during CPB between the groups (Fig. 1), consumptive coagulopathy as well as dilutional coagulopathy may underlie the development of hypofibrinogenemia during CPB in aortic replacement surgery.

There have been several reports of increased coagulation activation markers (e.g., prothrombin fragment 1 + 2, TAT, fibrinopeptide A, fibrin monomer) during CPB in CABG surgery [9]. In this study, significantly higher TAT levels during CPB were demonstrated in the aortic replacement group compared with the valve surgery group (Fig. 2). The TAT level, which reflects thrombin generation, was extremely high (i.e., the mean value was 180 ng/mL) at the end of CPB in the aortic replacement surgery group, as demonstrated by the representative case (Fig. 5b). This observation indicates the dramatic activation of coagulation during CPB in aortic replacement surgery because of the TAT level of approximately 40 ng/mL that is typical of disseminated intravascular coagulation caused by sepsis or hematological malignancy [10]. The significant elevation of TAT during CPB in aortic replacement surgery may reflect the CPB time. However, the significant difference in TAT levels were already observed at 1 h from the start of CPB in spite of no difference at the start of CPB between the groups, indicating that the elevation of TAT did not correlate with the CPB time in each group. Thus, thrombin generation was strikingly elevated in the aortic replacement group, independent of the CPB time. The TAT will reflect the generation of thrombin through the prothrombinase complex, and this reflects both extrinsic and intrinsic pathways, more correctly tenase pathways, with a complexity of positive and negative feedback pathways and regulatory processes which are no doubt highly activated during cardiac surgery. Thrombin generation during CPB might be influenced by the concentration of heparin, recirculation of blood aspirated from surgical field, and the coating of the extracorporeal circuit [11–14]. Previous analyses of thrombin generation and extrinsic coagulation pathway activation in suctioned blood from the pericardium and pleural space [15, 16] have demonstrated that the TAT level from the pleural cavity was much higher than that in the plasma. Thus, TAT elevation during CPB in aortic repair surgery may be primarily attributed to the recirculation of blood aspirated from the surgical field.

More importantly, the elevation of FVIIa was also significantly higher in the aortic replacement surgery group compared with the valve surgery group (Fig. 3), indicating a highly activated extrinsic coagulation pathway in the latter group despite full heparinization during CPB in both groups. Although heparin can strongly inhibit the intrinsic coagulation pathway as well as thrombin, its inhibitory effect in the extrinsic coagulation pathway may be relatively weak. Several reports, including the analysis on time course of intraoperative coagulation activation in elective CABG surgery [6], have demonstrated the activation of extrinsic coagulation pathway during CPB [12]. FVII is activated by TF that is released from tissues and/or the vascular injury site. As aortic replacement surgery is highly invasive, leaked blood into the pleural cavity in the surgical field may contain high amounts of TF. This leaked blood is usually recirculated into the CPB through suction, resulting in the accelerated activation of the FVII in the patients’ blood. Taken together, the consumptive coagulopathy due to activation of extrinsic coagulation pathway, may progress continuously during CPB in aortic replacement surgery. It has been suggested that reducing thrombin generation in CPB may result in decreased blood loss and transfusion volumes [12, 14, 17]. Managing the heparin concentration during CPB may contribute to the inhibition of coagulation activation during CPB [18, 19]. Heparin induces the release of the tissue factor pathway inhibitor (TFPI), which binds to TF and inactivates it [10], from vascular endothelial cells [20]. The increase in TFPI in heparinized blood during CPB [21] may suppress TF/FVIIa activity to some extent. In any case, consumptive coagulopathy may progress during CPB in aortic replacement surgery as shown in this study, and thus, it is useful to evaluate the change of the activation markers of coagulation during CPB.

Although we observed the tendency for larger increases in the SFMC during CPB in the aortic replacement group compared with the valve surgery group, the difference was not significant between the groups (Fig. 4). SFMC includes soluble fibrin and consists of one-molecule of fibrin monomer and two-molecules of fibrinogen, fibrin degradation products, and fibrinogen. This explains how the level of SFMC is influenced by fibrin degradation products and fibrinogen, but it does not necessarily reflect an increase in TAT. Moreover, the half-life of SFMC (i.e., a couple of hours) is much longer than that of TAT (i.e., less than half hour). In some cases of thoracic aortic aneurysm, considerably high levels of SFMC (e.g., >50 μg/mL) were detected before surgery. The SFMC values were influenced by not only the activation of coagulation pathway but also fibrinolytic pathway. Moreover, the size of aneurysm may influence the data of SFMC due to the activation of fibrinolytic system, resulting in the variable data of SFMC before surgery. This resulted in no significance in the SFMC elevation during CPB between the groups. Thus, elevation of SFMC in aortic replacement surgery may be influenced not only by intraoperative tissue injury, but also by preoperative progression of vascular atherosclerosis. Sustained elevation of SFMC after CPB suggests intravascular fibrin or microthrombi formation after CPB, contributing to organ dysfunction in patients who underwent aortic replacement surgery [22, 23].

## Conclusions

The activation of coagulation cascade as indicated by increases in FVIIa and increased thrombin generation in TAT during CPB was dramatically higher in the aortic replacement surgery group compared with the valve surgery group. This could potentially exacerbate consumptive coagulopathy during and after CPB in patients who underwent aortic replacement, possibly resulting in perioperative massive hemorrhage due to impaired hemostasis.

## Abbreviations

APTT: Activated partial thromboplastin time
AVR: Aortic valve replacement
CABG: Coronary artery bypass grafting
CPB: Cardiopulmonary bypass
FFP: Fresh frozen plasma
FVIIa: Activated factor VII
MVP: Mitral valve plasty
MVR: Mitral valve replacement
PC: Platelet concentrate
PT: Prothrombin time
RBC: Red blood cell
SFMC: Soluble fibrin monomer complex
TAA: Thoracic aortic aneurysm
TAP: Tricuspid annuloplasty
TAR: Total arch replacement
TAT: Thrombin–antithrombin complex
TF: Tissue factor
TFPI: Tissue factor pathway inhibitor
